# Editorial: A Journey Through 50 Years of Structural Bioinformatics in Memoriam of Cyrus Chothia

**DOI:** 10.3389/fmolb.2022.885318

**Published:** 2022-04-28

**Authors:** Alfredo Iacoangeli, Paolo Marcatili, Charlotte Deane, Arthur M. Lesk, Annalisa Pastore, Sarah A. Teichmann

**Affiliations:** ^1^ Department of Biostatistics and Health Informatics, King’s College London, London, United Kingdom; ^2^ Department of Basic and Clinical Neuroscience, King’s College London, Maurice Wohl Clinical Neuroscience Institute, London, United Kingdom; ^3^ King’s College London, National Institute for Health Research Biomedical Research Centre and Dementia Unit at South London and Maudsley NHS Foundation Trust King’s College London, London, United Kingdom; ^4^ Department of Bio and Health Informatics, Technical University of Denmark, Kongens Lyngby, Denmark; ^5^ Department of Statistics, University of Oxford, Oxford, United Kingdom; ^6^ Department of Biochemistry and Molecular Biology, The Pennsylvania State University, University Park, PA, United States; ^7^ European Synchrotron Radiation Facility, Grenoble, France; ^8^ Wellcome Sanger Institute, Hinxton, United Kingdom; ^9^ Theory of Condensed Matter Group, Cavendish Laboratory/Department of Physics, University of Cambridge, Cambridge, United Kingdom

**Keywords:** computational biology, bioinforamtics, structural bioinformatic, structural Biology, theoretical biology

Dr Cyrus Chothia FRS was a pioneer and one of the founding figures of theoretical and computational biology, nowadays commonly known as the field of bioinformatics (a term which Cyrus never quite got used to). To cite some of his numerous contributions to the field, the work of Cyrus and co-workers on the relationship between the divergence of sequence and divergence of structure in proteins supported the development of methods of homology modelling (Chothia and Lesk, 1986); his work on mechanisms of conformational change included one of the first characterizations of the structural differences between deoxy- and oxy-haemoglobin (Baldwin and Chothia, 1979; Lesk et al., 1985); his novel taxonomic approach to the study of the relationship between the sequence, structure, function, and evolution of proteins, led to the discovery of canonical structures of complementarity-determining regions (CDRs) of antibodies (Chothia and Lesk, 1987) and the creation of a first hierarchical classification of proteins into subfamilies, families and superfamilies based on structural, and functional similarities (Murzin et al., 1995). His seminal research had major impacts in the field during a long career of over 50 years, and remains a source of inspiration for new generations. We would like to honour his memory and pay tribute to his work with this Research Topic.

As mentioned above, Cyrus’ work included diverse computational research areas including antibody canonical loop classification. Two articles in this collection focus on the computational study and design of antibodies. Antibodies can be used to target toxic molecules. In their work, Gilodi et al. coupled experimental methodologies with in silicio design and showed the potential of developing an antibody able to recognize the RNA binding regions of TDP-43. TDP-43 aggregates have been proposed as a potential cause of ALS and their sequestration has been proposed as a therapeutic strategy.

Although the variable domains of an antibody contain the complementarity-determining regions (CDRs) that shape and host the antigen binding site (ABS), the elbow angle and the relative interdomain orientations of the variable and constant domains also influence the shape of ABS (Lesk and Chothia., 1988). Therefore, understanding the link between their dynamics and antigen specificity is crucial for the modelling and engineering of antibodies. Using molecular dynamics techniques, Fernández-Quintero et al. investigated this relationship and found that CDR loops reveal conformational transitions in the micro-to-millisecond timescale, while the interface and elbow angle dynamics occur on the nanosecond timescale.

The next three studies discuss and attempt to identify general features of protein domains, in the spirit of Cyrus’ analyses of protein structures and sequences. Chen et al. used a novel approach based on the study of domain-mediated protein-protein interactions, rather than a traditional focus on individual domains, for the structural profiling of bacterial effectors. These are proteins injected by the bacteria into the host cells that are critical for their virulence and intracellular survival. Their approach led to novel quantitative insights into the structural basis of effectors that might aid the design of effective and selective inhibitors of their pathogenic mechanisms.

As studied by Cyrus particularly in later years of his career, interconnected functional, biophysical, and structural constraints drive the purifying selection leading to variable levels of conservations along protein sequences. In their work, Dubreuil and Levy discussed these constraints while emphasising relevant works of Cyrus. Subsequently, they focused their attention on the evolutionary rate of disordered regions and the role of cellular abundance in their sequence conservation. They found that disordered regions are equivalent to super-accessible surface residues, and they confirmed the strong divergence interdependency between surface and core residues and the weak evolutionary coupling of disordered and domain regions. Finally, they observed that protein abundance impacts the conservation of residues in core, surface and disordered regions with constraints of similar effect size.

In the spirit of Cyrus’ global approach to analysing the protein Universe, Konagurthu et al. tried to answer the following question: ‘What is the architectural “basis set” of the observed Universe of protein structures?’ The authors used an information-theoretic inference method to identify automatically conserved sets of secondary structural elements within any given collection. By applying this method to the ASTRAL SCOP domains, they created an architectural dictionary of 1,493 substructures and used it to dissect the protein data bank (PDB). They made the entire dictionary, associated information and all the concept instances from the analysis of the PDB, publicly available on a webserver (http://lcb.infotech.monash.edu.au/prosodic).

Homology modelling is one of the most established approaches to protein structure prediction and a longstanding tool for Cyrus and co-workers on important structures such as the model of the T cell receptor based on antibody structures (Chothia et al., 1988). Homology models rely on the accurate identification of a suitable structural template based on the sequence of the target protein. Recently, deep learning has shown great potential to mine the coevolutionary information from multiple sequence alignments, leading a substantial improvement in the detection of distant homology. An amusing anecdote is that Cyrus had an antipathy towards the term “coevolution”. He correctly pointed out that substitutions are always sequential rather than simultaneous. In a mini-review, Bhattacharya et al. presented the current advances of the protein homology detection field driven by the use of machine learning in Inter-Residue Interaction Map Threading.

Some classes of proteins present a low sequence identity among homologs limiting the use of sequence-based methods for their homology modelling. G protein-coupled receptors (GPCRs) represent one such example. However, GPCR sequences with similar patterns of hydrophobic residues are often structural homologs, even with low sequence identity. In their study, Jabeen et al. designed a method for homology modelling of GPCRs that exploits this biophysical characteristic, as well as other GPCR-specific features. Their method was validated with a number of published benchmarking datasets and a case study on an olfactory receptor is presented in the article. Furthermore, it was implemented in the form of a free tool called Bio-GATS (https://github.com/amara86/Bio-GATS).

Savojardo et al. investigated whether and to what extent single-amino acid pathogenic variants (PVs) could be associated with their solvent exposure. Solvent-Accessible Surface Area (SASA) is indeed a key characteristic of proteins in determining their folding and stability. Savojardo et al. mapped PVs onto a curated set of structures and determined that PVs occur more frequently in residues which are less likely to be accessible by the solvent, and that they are not evenly distributed among the different residue types. Using an in-house deep learning method for the sequence-based prediction of residue SASA, the authors confirmed these results in 12,494 human protein sequences for which no 3-D structure was available.

On a related topic, Di Renzo et al. investigated the interactions between amino acids on the protein surface and the solvent (water) to characterise their solvation properties. Although many descriptors of such properties exist, the local environment of each residue in the context of the protein is complex and often overlooked by existing methods. Based on molecular dynamics simulations, Di Renzo et al. developed a method to characterize the dynamic hydrogen bond network at the interface between protein and solvent, from which they derive the solvation properties of each amino in the protein environment.

Finally, in their review Bordin et al. presented some of Cyrus’ accomplishments in the context of the history of protein structure classifications. The authors particularly focused on SCOP and CATH, two major protein structural classifications databases, and the evolutionary insights these two classifications have brought. They conclude their piece by discussing how the growing volume of data, and integration of protein sequences into these structural classifications, is helping to predict new functions in Metazoan organisms.

The articles in this collection cover very diverse areas of Structural Bioinformatics, reflecting the broad impact of Cyrus’ research.

As a final remark, Cyrus never forgot that one’s life is about the journey and not only the destination, and was ahead of his time in the open-minded way that he collaborated with scientists of all backgrounds and nationalities as well as across disciplines. The editors and authors of this collection express their deepest gratitude to Cyrus for his enormous contribution to the field of Bioinformatics and for being a generous, supportive, and inspiring colleague and friend.
